# Downturn in Childhood Bone Mass: A Cross‐Sectional Study Over Four Decades

**DOI:** 10.1002/jbm4.10564

**Published:** 2021-11-26

**Authors:** Björn E Rosengren, Erika Bergman, Jessica Karlsson, Henrik Ahlborg, Lars Jehpsson, Magnus K Karlsson

**Affiliations:** ^1^ Clinical and Molecular Osteoporosis Research Unit, Department of Clinical Sciences and Orthopedics Lund University, Skåne University Hospital Malmö Sweden

**Keywords:** SINGLE‐PHOTON ABSORBTIOMETRY, BMD, FRACTURE RISK ASSESSMENT, FRACTURE PREVENTION

## Abstract

Screen time and physical inactivity have increased among children. As physical activity is a determinant of bone mass, there is a concern that children today have lower bone mass than earlier. If this is true, fractures may become more common in the future. In 2017–2018, we used single‐photon absorptiometry (SPA) to measure distal forearm bone mineral density (BMD; mg/cm^2^) in a normative cohort of 238 boys and 204 girls aged 7 to 15 years. We compared these results to BMD in a normative cohort collected in 1979–1981 (55 boys and 61 girls aged 7 to 15 years) measured by the same scanner. To investigate difference between the two cohorts, we used multiple linear regression with age, sex, and cohort as predictors. Predicted bone density at age 16 years was estimated through the slope values. The bone density‐age slope was flatter in the cohort measured in 2017–2018 than in the cohort measured 1979–1981 (−5.6 mg/cm^2^/yr [95% confidence interval −9.6 to −1.5]). Predicted bone density was at age 16 years in 2017–2018 in boys was 10% lower (−0.9 SD) and in girls 11% lower (−1.1 SD) than in their counterparts measured in 1979–1981. We found indications that children nowadays develop lower bone mass than four decades ago, giving concern that they may have a higher risk of osteoporosis and fragility fractures as they grow old. © 2021 The Authors. *JBMR Plus* published by Wiley Periodicals LLC on behalf of American Society for Bone and Mineral Research.

## Graphical abstract

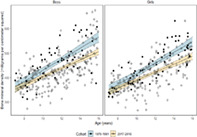

## Introduction

Thirty percent of children and 50% of women and 25% of men aged ≥50 years suffer fractures.^(^ ^1^, ^2^ ^)^ One of the strongest risk factors is low bone mass.^(^ ^3^ ^)^ Around 60% to 80% of the variance in bone mass is genetically determined, whereas the rest depends on lifestyle.^(^ ^4^ ^)^ Physical activity is one of the most important lifestyle factors for bone mass,^(^ ^4^ ^)^ and high physical activity is in all ages associated with high bone mass^(^ ^5^, ^6^ ^)^ and low fracture risk.^(^ ^3^, ^7^ ^)^

Bone mass increases during the first decades in life, and 25% of the adult bone mass is acquired during two pubertal years.^(^ ^8^ ^)^ Furthermore, the greatest skeletal response to mechanical load occurs in pre‐ and early pubertal years.^(^ ^9^ ^)^ High level of physical activity during growth is also associated with high bone mass at the end of growth, that is, peak bone mass,^(^ ^6^, ^10^ ^)^ but also in adulthood.^(^ ^10^, ^11^ ^)^ Peak bone mass is further estimated to predict 50% of the variance in bone mass at old age,^(12^ ^)^ and a 10% increase in peak bone mass delays the development of osteoporosis by 13 years.^(^ ^13^ ^)^ It is therefore possible that increased physical activity, initiated before or in early puberty, could counteract osteoporosis^(^ ^10^, ^11^ ^)^ and decrease fracture risk in adulthood.^(^ ^3^, ^7^, ^10^, ^11^ ^)^

Despite the above knowledge, physical inactivity has reached enormous proportions.^(^ ^14^, ^15^ ^)^ This type of inactivity may continue further as screen time activity through computers, tablets, and smart phones is increasing,^(^ ^14^ ^)^ nowadays covering as much as 40% to 60% of all sitting time for children.^(14^ ^)^ Also increasing is the proportion that uses screens ≥2 hours per day, rising from 15% to 35% in 2002 to 65% to 70% in 2014.^(^ ^14^ ^)^ This change has raised concern for lower childhood bone mass, lower peak bone mass, and a steep increase in number of fractures in the future. However, we are unaware of any study comparing bone mass in children from the same region born and raised before and after the widespread use of computers, smart phones, and tablets.

Based on the unanimous results regarding increased physical inactivity in children,^(^ ^14^, ^15^, ^16^ ^)^ we hypothesized that bone mass in a pediatric population today would be lower than four decades ago.

## Subjects and Methods

### Study participants

Malmö, the third largest city in Sweden, had a population of 235,111 (38,651 aged <16 years) in 1979.^(^ ^17^ ^)^ During 1979–1981, 116 children (55 boys and 61 girls) aged 7 to 15 years from the city, all White, partook in a study to determine normative data for pediatric bone mass.^(^ ^18^ ^)^ The study used single‐photon absorptiometry to measure bone mass in both distal forearms. The children were invited in a non‐population‐based approach. We have no information on how many children were invited but denied participation.

In 2017, the city population had increased to 333,633 (64,309 aged <16 years).^(^ ^17^ ^)^ During 2017–2018, we invited 976 children (491 boys and 485 girls) living in the city to have their distal forearms scanned by the same scanner that was used in 1979–1981. The children were all students at one of three government‐funded schools to which they were allocated according to their residential address. A total of 442 children (238 boys and 204 girls) aged 7 to 15 years, of whom 95% were White, accepted participation (45% attendance rate).

### Measurements

Forearm bone mineral density (mg/cm^2^) was measured 6 cm proximal to the styloid process of the ulna by single‐photon absorptiometry. We used a rectilinear scan across the radius and ulna, with a radiation source (241 Americium) and a detector moving simultaneously, according to the method of Nauclér and colleagues.^(^ ^19^ ^)^ Both the right arm and the left arm were scanned, and the mean values of both arms were used. If the participants had a history of fracture in one arm, we only measured the uninjured arm. We excluded scanned arms where the scan quality made adequate plotting impossible. By these criteria, we ended with 4 children measured in 1979–1981 and 14 children measured in 2017–2018 with data from only one forearm. Four children (3.4%) measured in 1979–1981 and 9 children (2.0%) measured in 2017–2018 had during the previous year had an arm fracture, thereby having only the unfractured arm scanned. We further had to exclude five arm scans because of technical measurement errors in children measured in 2017–2018, rendering the scan of only one arm being used in four (3.4%) of the children measured in 1979–1981 (two non‐dominant arms and two with unknown dominance) and 14 (3.2%) of the children measured in 2017–2018 (seven non‐dominant arms and seven dominant arms).

The same densitometer was used for measuring study participants in 1979–1981 and in 2017–2018. During the study period, long‐term drift was 0.1% per year (95% confidence interval [CI] −0.2 to 0.4) evaluated by a standardized phantom. In 1980, the radiation source was replaced. All measurements in this study were adjusted with respect to repeated phantom measurements. The precision (coefficient of variation) of the SPA measurements was 2.7% when determined by 311 standardized phantom measurements and 4.8% when determined by three repeated measurements of 20 different arms (after repositioning). One technician performed all measurements in 1979–1981 and two technicians in 2017–2018. All measurements followed the standard protocol for distal forearm bone scanning, and all were inspected and analyzed in random order by one of the researchers. We used standard equipment to evaluate weight and height in both 1979–1981 and 2017–2018. Body mass index was calculated as weight divided by height squared (kg/m^2^).

We used linear regression models, including group, sex, and age as predictors, to investigate difference (ie, if the slopes in relation to age differed) between the cohorts. The model fit is presented in scatter plots as linear slopes with 95% CIs. As some of the children were almost 16 years old at the time of measurement, we chose to present predicted sex‐specific bone density difference at age 16 years, estimated as the difference between the two slope values at this age. The estimated difference at age 16 years is reported as absolute difference, proportional difference, and difference expressed as standard deviations (SD). Normative forearm bone density values in men and women aged 30 to 45 years were retrieved from previously published normative data^(^ ^20^ ^)^ and used to express the difference at age 16 years in standard deviations. We regarded a *p* value of <0.05 as a statistically significant difference. Since the 1979–1981 cohort was already measured when this study was planned, the power calculations were to a large extent regulated by this sample size. However, to reach higher power, we invited all children in all classes in the three schools, rendering more than three times as many study participants in 2017–2018 as in 1979–1981. We used R version 4.0 and RStudio version 1.3 for statistical calculations. The Ethical Review Board in Lund, Sweden, approved the study (reference number 2016/1680). Written consent was obtained before study start from participants and parents/guardians of each participant.

## Results

**Fig. 1:**
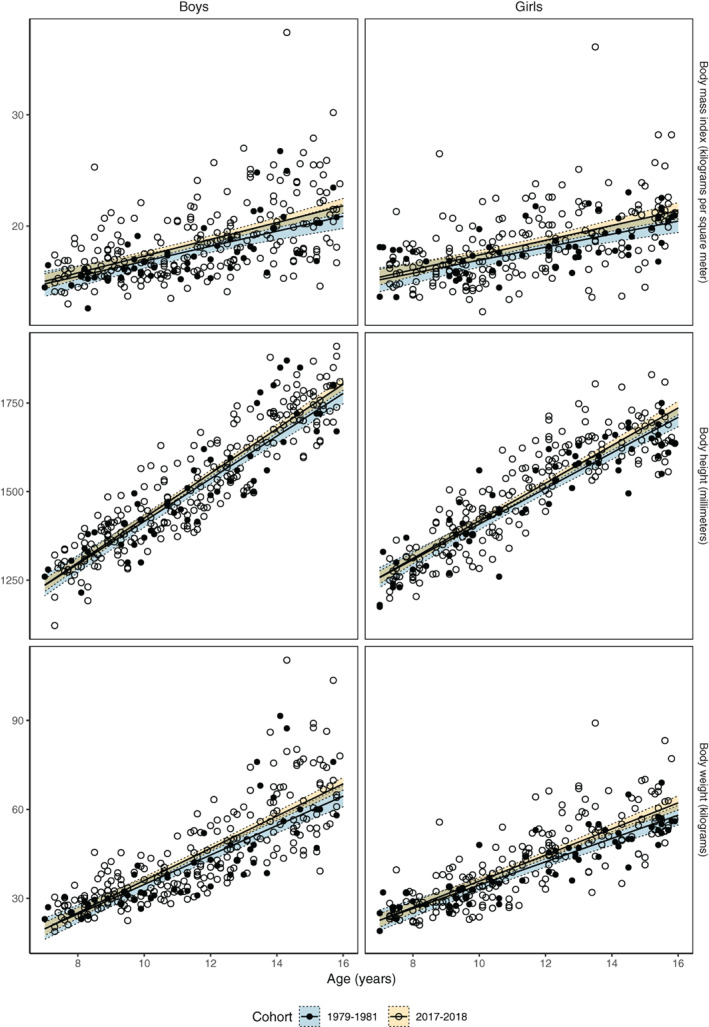
Anthropometric data (in relation to age) measured by standard equipment in two normative cohorts of boys and girls aged 7 to 15 years and measured in 1979–1981 and 2017–2018. Lines depict linear regression equations with 95% confidence interval bands fitted to the sex‐specific scatterplots.

Sex‐specific height, weight, and body mass index in relation to age for the 1979–1981 and the 2017–2018 cohorts are presented in Fig. 1 {FIG1} and sex‐specific height and weight in 3‐year age classes in relation to Swedish normative data in children born 1981^(^ ^21^ ^)^ in Table 1. {TBL 1}

**Table 1:** Height (cm) and Weight (kg) Presented in 3‐Year Age Classes in the Children Measured in 1979–1981 and 2017–2018 in Relation to Swedish Normative Data That Include Children Born in 1981^(^ ^21^ ^)^

	Girls			Boys		
	Ages 7–9 years	Ages 10–12 years	Ages 13–15 years	Ages 7–9 years	Ages 10–12 years	Ages 13–15 years
Body length (cm)						
Cohort measured in 1979–1981	131.8 (8.1)	150.4 (9.0)	162.8 (5.8)	135.1 (7.2)	148.6 (7.7)	169.6 (12.2)
Cohort measured in 2017–2018	133.6 (7.5)	150.7 (9.8)	165.1 (6.7)	134.6 (7.3)	150.8 (9.1)	171.5 (8.9)
Swedish children born in 1981	131.2	149.2	163.2	131.6	147.9	167.5
Body weight (kg)						
Cohort measured in 1979–1981	28.5 (3.9)	40.4 (6.7)	52.6 (6.4)	29.2 (4.6)	37.5 (6.3)	59.4 (15.7)
Cohort measured in 2017–2018	29.7 (6.2)	41.4 (9.3)	55.9 (10.6)	30.4 (5.8)	41.0 (8.4)	62.3 (14.3)
Swedish children born in 1981	28.8	41.0	54.9	29.0	40.2	57.1

When we evaluated traits as a function of age, we found similar results in children measured in 2017–2018 compared with 1979–1981 (reference group) for height (+0.3 cm per year [95% CI −0.3 to 0.9]), for weight (+0.5 kg per year [95% CI −0.2 to 1.2]), and for body mass index (+0.1 kg/m^2^ per year [95% CI −0.1 to 0.3]).

**Fig. 2:**
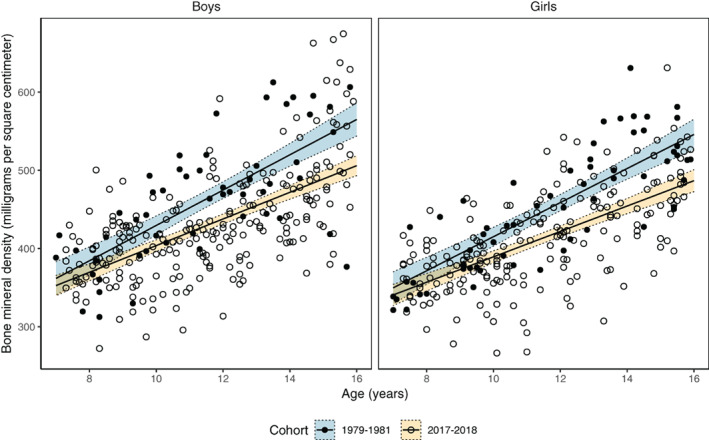
Distal forearm bone mineral density (mg/cm^2^) (mean value of right and left forearm) measured by single‐photon absorptiometry in two normative cohorts of boys and girls aged 7 to 15 years and measured in 1979–1981 and 2017–2018. Lines depict linear regression equations with 95% confidence interval bands fitted to the sex‐specific scatterplots.

Sex‐specific distal forearm bone density in relation to age for the 1979–1981 and the 2017–2018 cohorts are presented in Fig. 2. {FIG2} When we evaluated bone density as a function of age, we found a statistically significantly flatter inclination in children measured in 2017–2018 compared with 1979–1981 (reference group) (−5.6 mg/cm^2^ per year [95% CI −9.6 to −1.5]).

When we used the linear regression model to estimate distal forearm bone density at age 16 years, the predicted value was 56 mg/cm^2^ lower for boys and 61 mg/cm^2^ lower for girls measured in 2017–2018 than in 1979–1981. This suggests that in 2017–2018, boys aged 16 years had 10% (−0.9 SD) lower bone density than in 1979–1981. The corresponding difference for girls 2017–2018 was 11% (−1.1 SD) lower bone density than in 1979–1981.

Twenty‐three percent of the children measured in 1979–1981 (16% of the girls and 31% of the boys) and 19% of the children measured in 2017–2018 (15% of the girls and 23% of the boys) reported having had a fracture.

## Discussion

We found indications of an inferior bone mass development in children nowadays compared with four decades ago, with predicted bone mass values at age 16 years, in both boys and girls, being about 1.0 standard deviation lower than in 1979–1981.

Even if a change in bone mass within the population is low, this may lead to a significant effect in number of fractures. Studies evaluating time trends in adult bone mass have reported a decline from 2005 to 2014 in the US in both adults ≤50 years^(^ ^22^ ^)^ and in older individuals.^(^ ^23^ ^)^ We have been unable to identify studies that evaluate time trends in bone mass accrual during growth, data of great importance for future health care planning. The possible trends in bone density suggested by this study therefore highlight great concern for future fracture burden in society.

One of the strongest risk factors for fracture is low bone mass.^(^ ^3^ ^)^ One standard deviation lower bone mass, estimated by single‐photon absorptiometry or dual‐energy X‐ray absorptiometry, has been associated with a doubled fracture risk.^(^ ^3^, ^24^ ^)^ Low bone mass is also associated with a high fracture risk both in children^(^ ^25^ ^)^ and adults.^(^ ^3^, ^24^ ^)^ Studies have also found increasing time trends for pediatric fracture incidence: in Sweden with a higher incidence 2007 than 1998,^(^ ^26^ ^)^ in Japan with a higher incidence in 1999–2007 than 1979–1987,^(^ ^27^ ^)^ and in Australia with a higher incidence in 2015 than 2005.^(^ ^28^ ^)^ However, higher fracture risk is dependent on a variety of factors,^(^ ^3^, ^29^ ^)^ not only low bone mass. It is thus important to provide actual data on time trends in childhood bone mass and not draw conclusions regarding skeletal status based on only fracture incidence.

Our study design does not enable causal conclusions. We, however, speculate that the lower bone density found in children in 2017–2018 than in 1979–1981, at least partly, may be referred to changes in physical activity patterns. Physical activity is one of the strongest predictors of bone mass,^(^ ^4^ ^)^ and a recent increase in physical inactivity and sedentary screen time activities is well established.^(^ ^14^, ^15^, ^16^, ^30^ ^)^ The World Health Organization recommends children to engage in a minimum of 60 minutes of daily moderate and vigorous physical activity,^(^ ^31^ ^)^ with activities that strengthen muscle and bone at least three times per week.^(^ ^31^ ^)^ Only 10% to 20% of Swedish children aged 11 to 15 years, however, meet these recommendations,^(16^ ^)^ fairly similar to many other countries.^(^ ^15^ ^)^ If increased physical inactivity is involved in the downturn in bone density, it seems reasonable to believe that increased physical activity would be counteracting. This notion is supported by results from exercise intervention studies.^(^ ^5^, ^6^ ^)^ Interestingly, one of these studies, which followed bone density gain from ages 8 to 15 years in children who reached Tanner stage V, found that children with daily school physical activity gained 0.5 to 1.2 standard deviation higher bone density than children with physical activity only 1 to 2 lessons per school week.^(^ ^6^ ^)^ Furthermore, the annual fracture incidence at the end of the study was 50% of the expected.^(^ ^7^ ^)^ These findings point to that increased physical activity may result in benefits of clinical significance, with bone mass benefits of a magnitude similar to the difference at age 16 years that we found between the 1979–1981 and 2017–2018 cohorts. Furthermore, if our findings are the result of secular trends in physical activity, we and others could only speculate as to the future development of all other noncommunicable diseases that are associated with physical inactivity.^(^ ^30^ ^)^ However, we must also emphasize that we have only provided an observational study, without possibility to draw inferences regarding causality. Lower physical activity is only one plausible explanation, but other factors such as decreased calcium intake, changes in nutritional habits, increased intake of sweet soft drinks, and secular trends in anthropometry may all influence any trends in pediatric bone mass.

Study strengths include the use of the same scanner, phantom measurements to take long‐term apparatus drift into account, that a single researcher plotted all scans in random order, and that all children were from the same city. Study limitations include the cross‐sectional study design and the small sample size in 1979–1981. The children measured in 1979–1981 were not randomly selected, which may have introduced a risk of selection bias. However, as we found similar bone density in young children in 1979–1981 and 2017–2018 and more and more divergent bone density by increasing age, it seems probable that lifestyle differences in the two cohorts account for the findings. As most children were of White ethnicity and living in socioeconomic middle‐class areas, it is questionable if the results are transferrable to children with other ethnicity and/or living under other circumstances in other areas. Further limitations include lack of information on lifestyle factors, dietary intake, and pubertal stages of the children, data that could give indications why there was a different pattern in the bone mass versus age slopes in the cohorts. It would also have been advantageous to have evaluated bone mass in several anatomical regions and by dual‐energy X‐ray absorptiometry, but this technique was not available in 1979–1981. Studies have, however, shown that distal forearm bone density measurements by single‐photon absorptiometry and dual‐energy X‐ray absorptiometry are highly correlated and that both scanning techniques predict fracture similarly.^(^ ^3^, ^24^ ^)^

We found indication that children today develop lower bone mass than four decades ago. This indicates they may have a higher risk for osteoporosis and fragility fractures as they grow old compared with current adults. Our findings must be verified in other studies.

## Disclosures

All authors state that they have no conflicts of interest.

### Peer Review

The peer review history for this article is available at https://publons.com/publon/10.1002/jbm4.10564.

## Data Availability

The data that support the findings of this study are available on request from the corresponding author. The data are not publicly available due to privacy or ethical restrictions.
